# Early Morphokinetic Monitoring of Embryos after Intracytoplasmic Sperm Injection with Fresh Ejaculate Sperm in Nonmosaic Klinefelter Syndrome: A Different Presentation

**DOI:** 10.1155/2015/827656

**Published:** 2015-12-30

**Authors:** Ali Sami Gurbuz, Ahmet Salvarci, Necati Ozcimen, Ayse Gul Zamani

**Affiliations:** ^1^Department of Obstetrics and Gynecology, Novafertil IVF Center, Meram Yeni Yol No. 75, Meram, 42090 Konya, Turkey; ^2^Department of Urology, Novafertil IVF Center and Konya Hospital, Meram Yeni Yol No. 75, Meram, 42090 Konya, Turkey; ^3^Medicana Konya IVF Center, Medicana Konya Feritpaşa Mah., Gurz Sok. No. 1, Selçuklu, 42060 Konya, Turkey; ^4^Department of Medical Genetic, Meram Tip Faculty, Necmettin Erbakan Universıty, Yunus Emre Mah., Meram, 42060 Konya, Turkey

## Abstract

The patient was diagnosed with nonmosaic 47, XXY Klinefelter Syndrome with the AZF deletion absent and SRY+. The nonmosaic 47, XXY karyotype was confirmed on a skin biopsy chromosomal analysis. Using only ejaculate motile sperms, 11 oocytes underwent ICSI and were placed rapidly in a time lapse (Embryoscope ©) with a specific culture dish. Biopsies were performed on six embryos on the 3rd day, and numerical chromosomal abnormalities were observed using the FISH test before transfer. PGS results were normal in only two embryos with normal morphokinetics in the Embryoscope. For clinical confirmation of pregnancy, ultrasonographic examination was performed during the 7th week of pregnancy, and two gestational sacs and fetal heart beat were observed.

## 1. Introduction

Eighty percent of KS cases have 47, XXY karyotypes, termed the classical form, while 20% have the 46, XY/47, XXY mosaic form, a high degree of aneuploidy, and X chromosome structural abnormalities [1, 2]. In the nonmosaic type, viable births have been reported following intracytoplasmic sperm injection (ICSI) with ejaculate and testis sperm [3]. In various studies, increased chromosomal anomaly rates in embryos obtained from males with KS have been reported with aneuploidy screening (PGS). It was also noted that, in the follow-up of these embryos, results indicative of unfavorable prognosis were obtained from pronuclear morphology evaluation, suggesting that the children will be born with KS [4]. It was suggested that some previously unknown characteristics during incubation may be the decisive criteria for the prospect of pregnancy in studies on embryo development. Therefore, procedures following early development have been initiated to increase the chance of pregnancy in IVF-ICSI cycles using a time lapse imaging incubator system (time lapse = Embryoscope) [5, 6]. This system can be used to determine whether irregularly or rapidly dividing embryos with impaired morphokinetics can occur. Time lapse imaging can be used to monitor embryos without removing them from the incubator. It was suggested that time lapse is used as an alternative to PGS in young patients and those at a low risk of aneuploidy, since it can be used to track early embryo morphokinetics [5, 6].

In the present case of nonmosaic KS, pregnancy and live viable birth were obtained with fresh ejaculate sperm. As an initial example of KS, early embryo development was followed by a time lapse system and embryo morphokinetics were controlled. In addition, preimplantation genetic diagnosis (PGS) was performed on embryos prior to transfer, and PGS and time lapse techniques were compared for the detection of chromosome number abnormalities.

## 2. Case

Our patient was 34 years old. He had been married for 9 years. Based on semen analysis, the volume was determined to be 3.4 cc, with a concentration of 2 × 10^6^/mL, 73% immotility, and 99% sperm with head and neck anomalies. The patient was diagnosed with nonmosaic 47, XXY, KS (based on peripheral blood culture) with the AZF deletion absent and SRY+ (Figure 3). The nonmosaic 47, XXY karyotype was confirmed on a skin biopsy chromosomal analysis. His spouse was a 30-year-old healthy female. Her karyotype was normal (46XX). According to ISCN, 20 metaphases were analyzed with HRB banding technique [7].

The couple attempted pregnancy twice with no success at another IVF center with IVF-ICSI using fresh ejaculate sperm and classical embryo monitorization.

**Figure 1:**
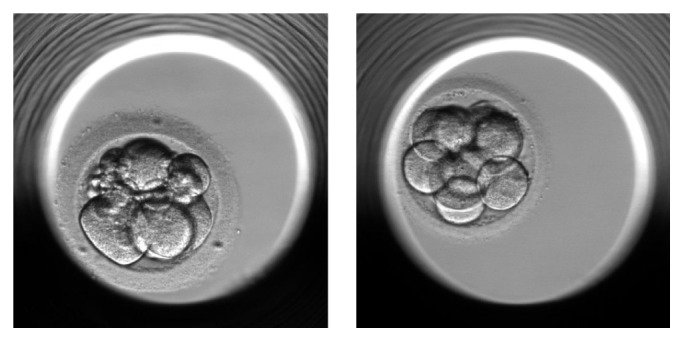
Two embryos: with normal morphokinetics in Embryoscope and with normal PGS (2nd and 6th embryos).

**Figure 2:**
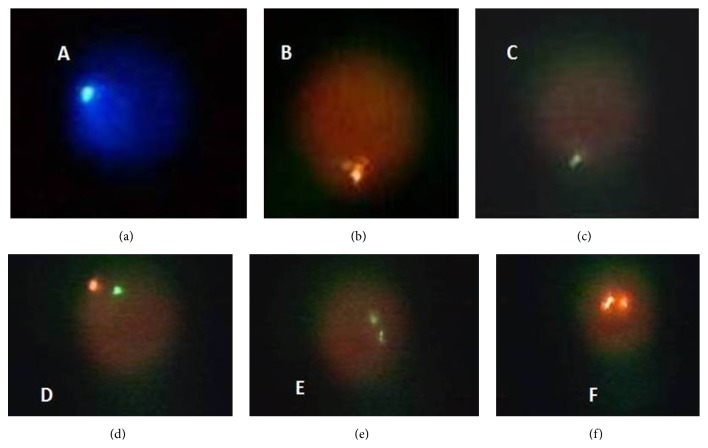
Different images of the patient's sperm FISH: (a) (18), (b) (Y), (c) (X) normal gametes, (d) (XY), (e) (XX), and (f) (YY) disomic gametes.

**Figure 3:**
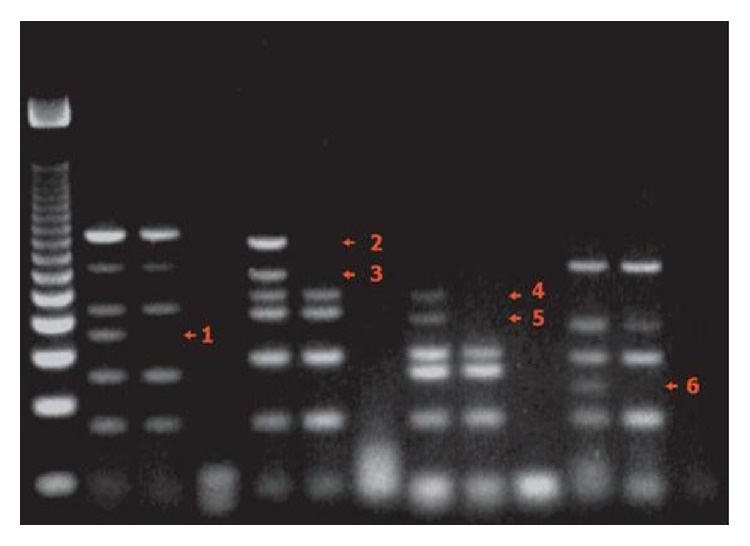
Agar gel, AZF with no deletions, and SRY (+).

The family was counseled regarding the probability of chromosomal number and structural abnormalities in an infant with KS, and embryonic monitorization and PGS were recommended. The family was also educated on the study and informed consent was obtained. Using only motile sperms, 11 oocytes underwent ICSI and were placed rapidly in an Embryoscope with a specific culture dish. Vitrolife sequential media were used for embryo culture, with embryos being cultured in G1 plus medium from days 0 to 3. Early morphokinetics of each embryo were followed by images obtained every 20 min with time lapse after ICSI [6, 8]. On the 2nd day of time lapse, pathological findings were observed in early embryo morphokinetics of seven embryos. In one embryo, total fertilization failure was observed. In the other two embryos, morphokinetics were normal. The time lapse until six embryos divided from 3 cells to 4 and 5 cells was 49 and 53 h, respectively, while the division times of two embryos with normal morphokinetics were found to be 24 and 31 h, respectively. Using the Vysis MultiVysion FISH probe, chromosome aneuploidy screening was performed on blastomeres using the multicolor FISH method (Figure 2). Biopsies were performed on six embryos on the 3rd day, and numerical chromosomal abnormalities were observed using the FISH test before transfer (monosomy 18, monosomy 21, trisomy 13, trisomy 21, XXY, and XXX) (Table 1). PGS results were normal in only two embryos with normal morphokinetics in the Embryoscope (Figure 1) and were transferred on the 5th day of oocyte retrieval. Twelve days following embryo transfer, hCG levels were measured as 782.75 pg/mL in blood. For clinical confirmation of pregnancy, ultrasonographic examination was performed during the 7th week of pregnancy, and two gestational sacs and fetal heart beat were observed. On the 37th week, a boy with a weight of 2,425 g and length of 48 cm and a girl with a weight of 2,812 g and length of 50 cm were delivered via Cesarean section. Peripheral leukocyte chromosome analysis of the infants revealed 46, XX and 46, XY karyotypes.

## 3. Discussion

In 1959 it was shown that KS is a chromosomal disease, and an extra X chromosome leads to this clinical presentation [1]. Between 1997 and 2013 pregnancy and births were reported in nonmosaic KS cases following ICSI with testicular sperm [4, 9]. Based on sperm analysis of nonmosaic KS patients, haploid sperms were observed at rates of 76.47% and 92.25% [4]. Based on FISH analysis, 91.38% of sperms had a haploid structure. PGS is recommended in IVF-ICSI on patients with KS using testicular or ejaculate sperms due to the higher rate of aneuploid chromosome abnormalities caused by gametes. In addition, it is believed that embryo scoring and selection should be performed according to PGS chromosome abnormality and pronuclear morphology, so that the chance of pregnancy is increased [4, 9]. In 1996 and 2000, Staessen and Bielanska et al., respectively, performed and recommended embryo biopsy for X and Y chromosomes in ICSIs with sperms obtained from patients with KS and suggested that, in the embryos of these patients, chaotic chromosome patterns would be present at a rate of 70% [9].

The time lapse imaging system is used for early embryo morphokinetics to select high quality embryos and increase the pregnancy rate to 0.22. Since the time lapse imaging system is a noninvasive method, it was proposed that it can be used in young patients and those at a low risk of aneuploidy instead of PGS to avoid embryo biopsy [6, 8]. We evaluated all embryos with time lapse and PGS. It was possible to see the embryos with the best morphokinetics with the contribution of time lapse which is currently believed to follow up early embryo development most objectively. If the family had not wanted PGS and if we had not decided to perform PGS, we would have used the embryos with the best morphokinetics observed with time lapse in the transfer.

## 4. Conclusion

Twin pregnancy and viable live birth were obtained in a patient with nonmosaic KS with embryos of sound structure and number using fresh ejaculate sperm. We have not decided relying only time lapse. Time lapse is a new application. The outcome even in the normal patients is still a wonder. Time lapse is currently the most objective method for monitoring embryo. We believe that there should be larger case studies including the monitoring of PGS and time lapse of both normal patients and patients with KS. Klinefelter group should be followed up so that healthy embryos can be selected by time lapse.

## Figures and Tables

**Table 1:** Result of PGS by FISH examination.

Embryo	13	18	21	XY	Result
1	2	1	1	N	Monosomy 18, monosomy 21
2	2	2	2	N	Normal
3	3	2	3	XXY	Trisomy 13, 21, XXY
4	3	2	2	N	Trisomy 13
5	2	1	2	XXX	Monosomy 18, XXX
6	2	2	2	N	Normal

PGS method: multicolor FISH; material used: blastomere; protocol number: PGT14-18; probes used: Vysis MultiVysion PGS FISH.

## References

[1] Lanfranco F., Kamischke A., Zitzmann M., Nieschlag P. E. (2004). Klinefelter's syndrome. *The Lancet*.

[2] Bojesen A., Juul S., Gravholt C. H. (2003). Prenatal and postnatal prevalence of Klinefelter syndrome: a national registry study. *Journal of Clinical Endocrinology and Metabolism*.

[3] Fullerton G., Hamilton M., Maheshwari A. (2010). Should non-mosaic Klinefelter syndrome men be labelled as infertile in 2009?. *Human Reproduction*.

[4] Kahraman S., Findikli N., Berkil H. (2003). Results of preimplantation genetic diagnosis in patients with Klinefelter's syndrome. *Reproductive BioMedicine Online*.

[5] Swain J. E. (2013). Could time-lapse embryo imaging reduce the need for biopsy and PGS?. *Journal of Assisted Reproduction and Genetics*.

[6] Freour T., Lammers J., Splingart C., Jean M., Barriere P. (2012). Time lapse (Embryoscope) as a routine technique in the IVF laboratory: a useful tool for better embryo selection?. *Gynecologie Obstetrique Fertilite*.

[7] Simons A., Shaffer L. G., Hastings R. J. (2013). Cytogenetic nomenclature: changes in the ISCN 2013 compared to the 2009 edition. *Cytogenetic and Genome Research*.

[8] Meseguer M., Herrero J., Tejera A., Hilligsøe K. M., Ramsing N. B., Remoh J. (2011). The use of morphokinetics as a predictor of embryo implantation. *Human Reproduction*.

[9] Greco E., Scarselli F., Minasi M. G. (2013). Birth of 16 healthy children after ICSI in cases of nonmosaic Klinefelter syndrome. *Human Reproduction*.

